# The Biocorrosion of a Rare Earth Magnesium Alloy in Artificial Seawater Containing *Chlorella vulgaris*

**DOI:** 10.3390/ma18153698

**Published:** 2025-08-06

**Authors:** Xinran Yao, Qi Fu, Guang-Ling Song, Kai Wang

**Affiliations:** 1 Department of Ocean Science and Engineering, Southern University of Science and Technology, Shenzhen 518055, China; yaoxr@mail.sustech.edu.cn; 2 Division of Materials Engineering, School of Mechanical and Mining Engineering, The University of Queensland, St Lucia, QLD 4072, Australia; 3 Dekai Intelligent Casting Co., Ltd., Zhuozhou 072750, China; wangkai483@aliyun.com

**Keywords:** Mg alloy, *Chlorella vulgaris*, biomineralized film, biocorrosion

## Abstract

In the medical field, magnesium (Mg) alloys have been widely used due to their excellent antibacterial properties and biodegradability. However, in the marine environment, the antibacterial effect may be greatly attenuated, and consequently, microorganisms in the ocean are likely to adhere to the surface of Mg alloys, resulting in biocorrosion damage, which is really troublesome in the maritime industry and can even be disastrous to the navy. Currently, there is a lack of research on the biocorrosion of Mg alloys that may find important applications in marine engineering. In this paper, the biocorrosion mechanism of the Mg alloy Mg-3Nd-2Gd-Zn-Zr caused by *Chlorella vulgaris* (*C. vulgaris*), a typical marine microalga, was studied. The results showed that the biomineralization process in the artificial seawater containing a low concentration of *C. vulgaris* cells was accelerated compared with that in the abiotic artificial seawater, leading to the deposition of CaCO_3_ on the surface to inhibit the localized corrosion of the Mg alloy, whereas a high concentration of *C. vulgaris* cells produced a high content of organic acids at some sites through photosynthesis to significantly accelerate the surface film rupture at some sites and severe localized corrosion there, but meanwhile, it resulted in the formation of a more protective biomineralized film in the other areas to greatly alleviate the corrosion. The contradictory biocorrosion behaviors on the Mg-3Nd-2Gd-Zn-Zr alloy induced by *C. vulgaris* were finally explained by a mechanism proposed in the paper.

## 1. Introduction

Magnesium (Mg) alloys, with their advantages of high specific strength, specific stiffness, and excellent biocompatibility, have exhibited broad application prospects in many fields such as automobiles, aerospace, marine engineering, and medicine [1,2,3,4,5,6]. However, their practical application in marine environments faces severe challenges because the marine environment, characterized by high salinity, high humidity, and abundant microorganisms, is highly complex, which makes Mg particularly susceptible to corrosion [7,8,9,10,11]. Seawater contains a high concentration of chloride ions that can disrupt the inherently loose and porous oxide film on the surface of Mg alloys, leading to severe localized corrosion [12].

It is quite challenging to protect Mg alloys from corrosion attack, especially their stubborn galvanic corrosion in engineering [13,14]. Even worse, the ocean harbors a diverse range of organisms, including bacteria [15,16,17], algae [18,19,20], and macrofouling organisms [21]. Previous studies have shown that microorganisms can attach and grow on the surface of metals, forming biofilms and accelerating the corrosion of carbon steels [22,23,24], stainless steels [25,26,27], and copper alloys [28,29,30] through two possible ways: (1) microbial metabolic activities producing acidic or other corrosive substances to lower the pH and enhance the corrosivity of the local environment within biofilm and (2) the biofilms impeding oxygen diffusion and thus forming oxygen-concentration-difference cells to trigger more severe localized corrosion.

Microorganism-induced corrosion, including bacterial corrosion, such as sulfate-reducing bacteria (SRB) and nitrate-reducing bacteria (NRB), can occur under different conditions, and its control is also difficult [31,32,33,34,35,36]. It has been found that certain algae can secrete organic substances during their growth, such as polysaccharides and proteins, forming an organic film on Mg alloys to alter the electrochemical properties [37]. Additionally, algal photosynthesis may also modify the dissolved oxygen concentration and pH of the surrounding environment, which means that under light conditions, algae may produce oxygen through photosynthesis, increasing dissolved oxygen levels locally, whereas in darkness, algal respiration may consume oxygen and release carbon dioxide, leading to a decrease in local pH. Such periodic fluctuations in dissolved oxygen concentration and pH will exert a complex influence on the corrosion behavior of the Mg alloy. However, research on algal-induced corrosion remains relatively scarce, particularly on Mg alloys, let alone the systematic and in-depth studies on the specific mechanisms, influencing factors, and effective mitigation strategies.

Mg alloys also have advantageous biocompatible and antibacterial properties. In the medical field, Mg alloys have found some applications as biodegradable orthopedic implants and cardiovascular stents because of their great biocompatibility [38,39,40,41]. They can also be made into medical tools due to the antibacterial performance stemming from the release of Mg^2+^ and surface alkalization of Mg, which can disrupt the integrity of cell membranes, inhibit enzymatic activity, and thereby impede microbial growth [41]. However, in the marine environment, Mg alloys may not exhibit antibacterial behavior, as their dissolved Mg^2+^ can either be quickly diluted or react with other ions in seawater, making it difficult to achieve an effective antibacterial concentration [9]. Moreover, marine microorganisms, protected by biofilms, may become more tolerant to Mg^2+^ [6]. More complicatedly, the marine environment hosts a far more diverse microbial community than the human body. Thus, some single-mechanism antimicrobial strategies may become ineffective against the marine organisms with a multi-microbial community. It is of scientific importance to know whether medical antibacterial Mg alloys can still exhibit an algicidal effect in marine environments. Given the significant structural and physiological differences between algae and bacteria, the bacterial sterilization mechanism of Mg may not be directly applicable to algae, and the antibacterial properties of Mg alloys in medical applications cannot be simply extended to marine environments either. Therefore, it is also of engineering significance to carry out in-depth research to tailor specific conditions for practical applications of Mg alloys in marine environments.

A thorough investigation has been conducted into the effect of *Chlorella vulgaris* (*C. vulgaris*) on the biofouling and corrosion behavior of WE43 and AM60 Mg alloys [42]. The results have demonstrated that the presence of *C. vulgaris* can significantly accelerate the corrosion of conventional Mg alloys WE43 and AM60, which can be ascribed to the *C. vulgaris* continuously secreted organic acids that chemically dissolve the surface film more rapidly. However, these two typical Mg alloys are mainly used in atmospheric conditions. Recently, a new rare-earth containing Mg alloy, Mg-3Nd-2Gd-Zn-Zr, was developed for marine applications. Apart from the corrosion resistance, the biocorrosion performance is also critical to the success of this alloy. The Mg-3Nd-2Gd-Zn-Zr alloy was developed primarily for the use in marine environments, not for medical applications. This paper intends to investigate its algicidal property and biocorrosion in marine environments, which is to some extent relevant to the antibacterial performance of Mg. Hopefully, in the future, this alloy may also find applications in the medical field. Therefore, the study reported in this paper employed scanning electron microscopy (SEM), electrochemical techniques, and X-ray photoelectron spectroscopy (XPS) to conduct an in-depth investigation of the alloy’s biocorrosion behavior in marine environments, aiming to further elucidate the interaction between microalgae and the Mg alloy and provide both scientific foundation and engineering guidance for the development of high-performance Mg alloys in marine engineering.

## 2. Materials and Methods

### 2.1. Sample Preparation

The Mg alloy employed in this study was custom-formulated Mg-3Nd-2Gd-Zn-Zr alloys, with precise elemental composition detailed in Table 1. The samples were precision-machined into 10 mm × 10 mm × 7 mm coupons and subsequently encapsulated in epoxy resin (3150-AB, Yasong, Hangzhou, China), leaving an exposed surface area of 10 mm × 10 mm for electrochemical and immersion testing. Prior to these tests, all the exposed working surfaces were sequentially ground using SiC abrasive papers (up to 1000 grit), ultrasonically cleaned in anhydrous ethanol, and then air-dried under ambient condition. All reagents and chemicals in this study were of analytical reagent (AR) grade.

### 2.2. Algal Culture

The *C. vulgaris* employed in the study was commercially purchased from Shanghai Guangyu Biological Technology Co., Ltd. (Shanghai, China), which was cultured in the F/2 solution. The F/2 solution was autoclaved at 121 °C for 20 min for sterilization beforehand. The compositions of the F/2 solution and simulated seawater were presented in the previous study [42]. To systematically evaluate the impact of *C. vulgaris* on the corrosion behavior of the Mg-3Nd-2Gd-Zn-Zr alloy, three different immersion conditions were established in (1) abiotic solution containing 100% of the sterilized F/2 solution, (2) biotic solution 1 comprising 90% (vol.%) of the sterilized F/2 solution and 10% (vol.%) of freshly prepared *C. vulgaris* inoculum, and (3) biotic solution 2 consisting of 90% (vol.%) of the sterilized F/2 solution and 10% (vol.%) of *C. vulgaris* inoculum that had been pre-cultured for 7 days. Fourteen-day immersion experiments were carried out at 23 ± 1 °C under a 12 h/12 h light–dark cycle. To characterize the growth trend of the *C. vulgaris* cells, the concentration variation with time was monitored using a spectrophotometer at a wavelength of 686 nm.

### 2.3. Electrochemical Measurements

Electrochemical tests were performed using a multi-channel electrochemical workstation (CS310X, Corrtest Instruments, Wuhan, China) configured with a conventional three-electrode system. The working electrode was made of the Mg-3Nd-2Gd-Zn-Zr alloy with exposed surface area of 1 cm^2^. A saturated calomel electrode (SCE) served as the reference electrode, while a platinum plate (10 × 10 mm × 1 mm) functioned as the counter electrode. Electrochemical impedance spectroscopy (EIS) measurements were conducted over a frequency range from 10^5^ to 10^−2^ Hz with an applied perturbation amplitude of 10 mV. Potentiodynamic polarization curves were subsequently measured from −0.5 V to +0.7 V versus open circuit potential (OCP) at a scan rate of 0.5 mV·s^−1^.

### 2.4. Surface Characterization

After 14-day immersion in the inoculated solution, the Mg alloys were visualized under a fluorescence microscope to detect the chloroplast autofluorescence from the algal cells attached on the surfaces. The Mg alloy samples were then immersed in phosphate buffered saline (PBS) containing 4% glutaraldehyde for 8 h to fix the biofilm on the surface. Dehydration was performed through sequential 10 min immersion one after one in 25%, 50%, 75%, and 100% (*v*/*v*) ethanol solutions. Finally, the samples were air-dried under a stream of cold air [43]. For the samples after immersion in the abiotic solution, ethanol rinsing and cold-air drying were directly performed.

Morphological analysis of the biofilms and corrosion products on both abiotic and biotic samples was conducted using SEM (PHENOM XL, Phenom World, Eindhoven, The Netherlands), while energy dispersion spectrometer (EDS) was employed to determine elemental composition. X-ray diffraction (XRD, MiniFlex 300, Rigaku, Tokyo, Japan) was utilized to analyze the phase composition of biofilms and corrosion products, with a scanning range of 5–90° (2θ) at a rate of 10° min^−1^. XPS tests (Thermo Fisher Scientific Nexsa, Waltham, MA, USA) were employed to determine the chemical valences of Mg, Ca, and O within the corrosion products formed on Mg alloy samples.

The corrosion products on the Mg-3Nd-2Gd-Zn-Zr alloy samples were removed according to ASTM G1-03 standard [42] using a descaling solution (200 g/L chromium trioxide, 10 g/L silver nitrate, 20 g/L barium nitrate in deionized water), followed by ethanol rinsing and air-drying under a cold air stream. After descaling, the surface morphologies of the corroded samples were characterized by SEM again. The corrosion penetration depth and width on the Mg alloy surfaces were quantitatively analyzed using a digital optical microscope (Olympus DSX1000, Olympus, Tokyo, Japan) and white light interferometer (SuperView W, CHOTEST, Shenzhen, China).

## 3. Results and Discussion

### 3.1. Electrochemical Results

**Figure 1:**
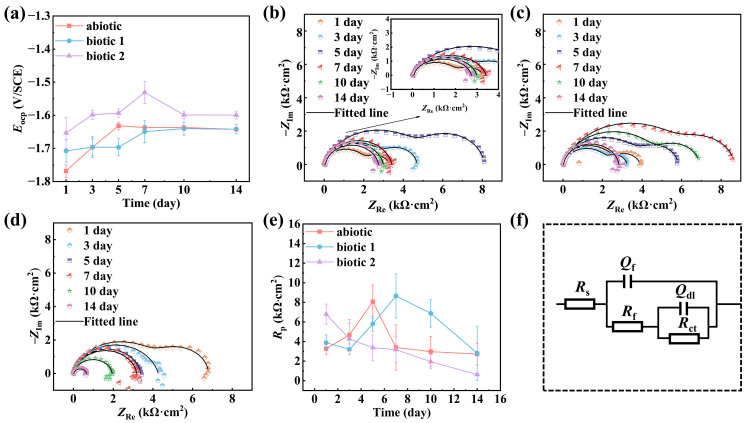
OCPs (**a**); Nyquist plots of Mg-3Nd-2Gd-Zn-Zr alloy in abiotic solution (**b**), biotic solution 1 (**c**), and biotic solution 2 (**d**) during 14-day immersion; and *R*_p_ values (**e**) obtained via curve-fitting based on an equivalent circuit (**f**).

Figure 1 shows the time-dependent OCP values, EIS spectra, and curve-fitted *R*_p_ results for Mg-3Nd-2Gd-Zn-Zr alloy in both the abiotic and biotic solutions throughout the 14-day light–dark cyclic immersion period. The OCP values shown in Figure 1a demonstrated a general positively shifting trend. In biotic solution 2, the significantly nobler potentials could be attributed to the cumulative effects of biofilm formation and corrosion product deposition. The Nyquist plots in all the solutions initially displayed two capacitive arcs (the high-frequency capacitive arc corresponding to the corrosion product layer and the low-frequency capacitive arc representing the charge transfer process), indicating that the initial surface films were intact. The alloy in the abiotic solution could experience a film rupture after 5 days of immersion, as its EIS exhibited a transition to single capacitive arc. Notably, in biotic solution 1, the EIS maintained two capacitive arcs on day 14, suggesting that the film did not break down in the first 13 days, and the film stability could be enhanced by algal-mediation through a biofilm barrier or biomineralization effect. In contrast, the alloy in biotic solution 2 demonstrated premature failure characteristics, showing single capacitive arc behavior on day 3 due to severe localized corrosion. 

The equivalent circuit shown in Figure 1f is used to fit the Nyquist plots of the Mg-3Nd-2Gd-Zn-Zr alloy in the abiotic and biotic solutions, where *R*_s_ represents the solution resistance; *Q*_f_ and *R*_f_ represent the capacitance and resistance of the surface film, respectively; and *Q*_dl_ and *R*_ct_ represent the double-layer capacitance and charge transfer resistance. Capacitance (*C*) is replaced with the constant phase element (*Q*). The impedance of *Q*_dl_ and *C*_dl_ can be calculated by Equation (1):

(1) $$Z_{\mathrm{CPE}}=Y_{0}^{-1}{\left(j\omega \right)}^{-n}$$

where *Y*_0_ and *n* are CPE parameters related to capacitance and surface heterogeneity and *j* and *ω* are the imaginary root and the angular frequency, respectively.

The derived electrochemical parameters from curves fitting are summarized in Table 2. The estimated *R*_p_ (=*R*_ct_ + *R*_f_) values for three solutions are compared in Figure 1e. The alloy in the abiotic solution showed an increase in *R*_p_ values during the first 5 days followed by a progressive decrease, which was consistent with the observed transition from dual to single capacitive arcs in Nyquist plots, confirming that the initially formed protective film subsequently broke down on the 5th day. In the biotic solution 1, the *R*_p_ value reached its maximum value within 7 days and then declined, approaching the level close to that in the abiotic solution finally on day 14 (i.e., the rupture of surface film might not occur in the first 13 days). This observation demonstrated that a low concentration of *C. vulgaris* cells could provide a certain inhibitive effect on the alloy’s corrosion. However, in biotic solution 2, the alloy’s *R*_p_ value progressively decreased throughout the 14-day immersion experiment, indicating that a high concentration of *C. vulgaris* cells could continuously weaken the corrosion resistance of the alloy (in this case, the rupture of the surface film could occur as early as day 3).

**Figure 2:**
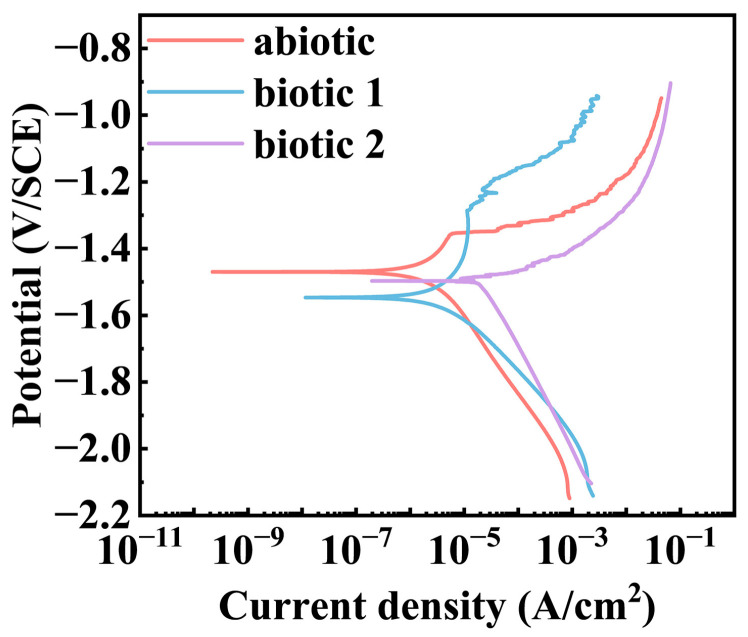
Potentiodynamic polarization curves of Mg-3Nd-2Gd-Zn-Zr alloy after 14 days of immersion in abiotic and biotic solutions.

Figure 2 presents the potentiodynamic polarization curves of the Mg-3Nd-2Gd-Zn-Zr alloy after 14-day immersion in the abiotic and biotic solutions. A comparative analysis demonstrated that the alloy in biotic solution 1 exhibited suppressed anodic dissolution in a positive potential range but noticeably increased anodic current density at potentials near the OCP and obviously accelerated cathodic process in a wide cathodic potential range relative to the polarization curve in the abiotic solution. The alloy in biotic solution 2 showed significantly enhanced anodic and cathodic processes. The curve-fitting parameters of the polarization curves after 14 days of immersion (Table 3) indicated that the lowest corrosion current density was only 2.29 μA/cm^2^ in the abiotic solution, while that in biotic solution 1 was higher, around 7.14 μA/cm^2^, and in biotic solution 2, the corrosion current density increased to 26.36 μA/cm^2^, one order of magnitude higher than that in the abiotic solution. These results confirmed that the high concentration of *C. vulgaris* cells dramatically accelerates the corrosion process of the Mg-3Nd-2Gd-Zn-Zr alloy in simulated seawater, further supporting EIS results.

### 3.2. Biofilm and Corrosion Product Film

**Figure 3:**
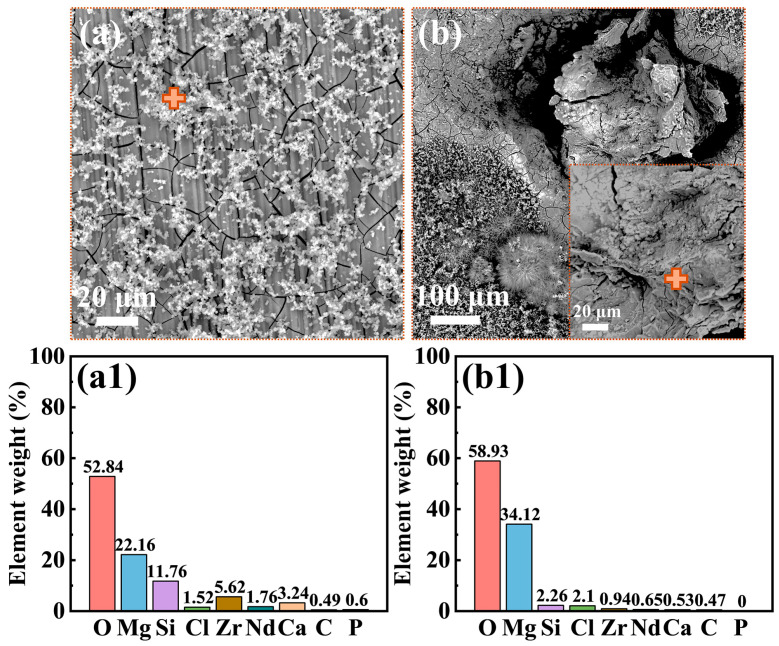
Morphologies of the surface films: (**a**,**b**) SEM images and (**a1**,**b1**) EDS results from the “+” areas for the Mg-3Nd-2Gd-Zn-Zr alloy after 14 days of immersion in the abiotic solution.

**Figure 4:**
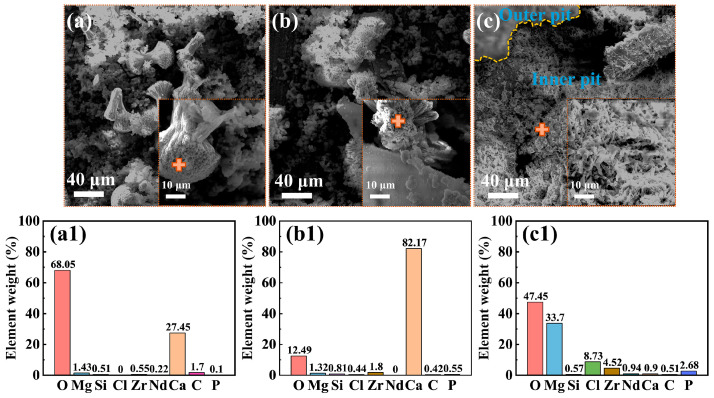
Morphologies of the surface films: (**a**) SEM images and (**a1**) EDS results from the “+” areas for the Mg-3Nd-2Gd-Zn-Zr alloy after 14 days of immersion in biotic solution 1; (**b**,**c**) SEM images and (**b1**,**c1**) EDS results from the “+” areas in biotic solution 2.

Figure 3 presents the morphologies of the corrosion product film and the EDS analysis results (marked by “+” signs) of the film on the Mg-3Nd-2Gd-Zn-Zr alloy after 14 days of immersion in the abiotic solution. As shown in Figure 3a, the intact regions of the alloy surface exhibited a dense, lamellar-structured corrosion product layer at the bottom, overlaid by loosely deposited corrosion products. The EDS analysis in Figure 3(a1) showed that this corrosion product film primarily consisted of O, Mg, Si, and Zr elements, with trace amounts of Cl, Nd, and Ca elements. Figure 3b shows localized ruptures of the film, where the corrosion products were mainly composed of Mg and O. Figure 4 displays the biofilm morphologies and EDS results for the Mg-3Nd-2Gd-Zn-Zr alloy after 14 days of immersion in biotic solutions 1 and 2. Figure 4b,(b1) presented abundant algal cells and extracellular polymeric substances (EPS) on intact surface areas, primarily containing O, Mg, and Ca elements. The elevated Ca content likely resulted from biomineralization by *C. vulgaris*. Figure 4c reveals more severe film ruptures in biotic solution 2 than in the abiotic solution. The corrosion products at a rupture site consisted mainly of O, Mg, and Zr elements, and notably, a relatively high concentration of Cl^−^ was detected there, suggesting that there was possibly a synergistic action between Cl^−^ and *C. vulgaris* to promote the film rupture. In summary, these results suggested that the metabolic products from low-concentration algal cells in biotic solution 1 might exhibit a certain inhibitive effect on the film rupture on the Mg-3Nd-2Gd-Zn-Zr alloy. Conversely, the high concentration of algal cells in biotic solution 2 significantly accelerated the film rupture, and why it had a different effect on the film rupture will be interpreted based on more experimental results later.

**Figure 5:**
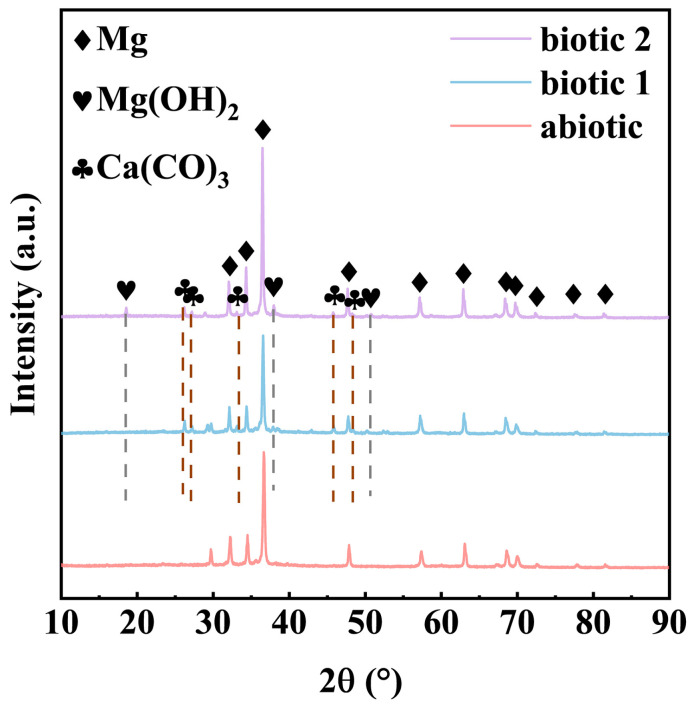
XRD spectra of Mg-3Nd-2Gd-Zn-Zr alloy after 14 days of immersion in abiotic solution, biotic solution 1, and biotic solution 2.

Figure 5 presents the XRD analysis results of the corrosion products formed on the Mg-3Nd-2Gd-Zn-Zr alloy surface in the abiotic and biotic solutions, and the XRD data were analyzed using the ICDD PDF database. The sample in the abiotic solution exhibited exclusively Mg peaks, suggesting the formation of an ultrathin corrosion product layer below the detectable threshold of XRD. The sample immersed in biotic solution 1 demonstrated an additional peak corresponding to crystalline CaCO_3_, confirming the CaCO_3_ precipitation through the algal biomineralization process. Immersion in biotic solution 2 resulted in a more complex phase composition, featuring three distinct crystalline components: the substrate Mg, CaCO_3_, and Mg(OH)_2_. The distinct phase evolution patterns of the films provided indirect evidence of concentration-dependent algal influences on the composition of corrosion products. The low concentration of *C. vulgaris* cells primarily led to protective CaCO_3_ deposition, while the high concentration of *C. vulgaris* cells promoted both the biomineralization and formation of detrimental organics (such as organic acids) through complex biochemical actions.

**Figure 6:**
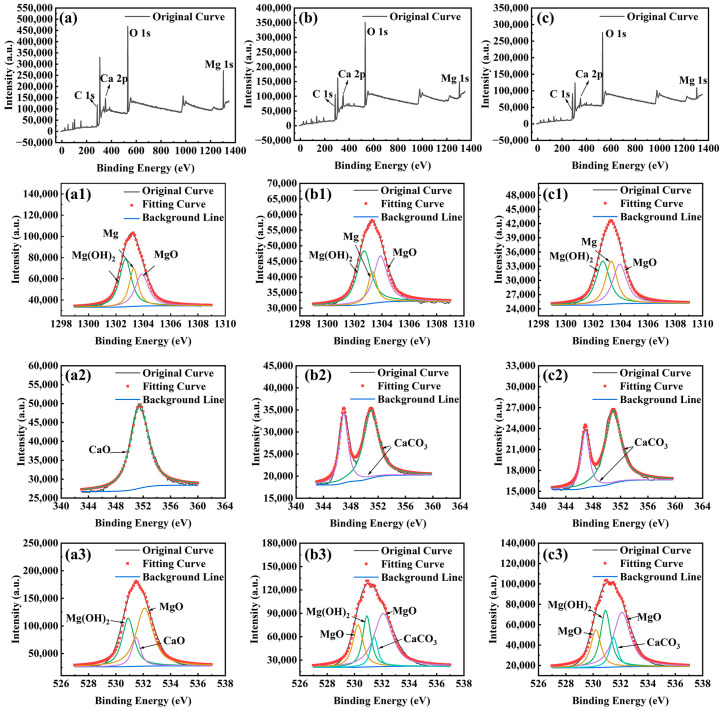
High-resolution XPS spectra for Mg-3Nd-2Gd-Zn-Zr alloy after 14 days of immersion in abiotic solution: (**a**) survey spectra, (**a1**) Mg 1s, (**a2**) Ca 2p, and (**a3**) O 1s; in biotic solution 1: (**b**) survey spectra, (**b1**) Mg 1s, (**b2**) Ca 2p, and (**b3**) O 1s; and in biotic solution 2: (**c**) survey spectra, (**c1**) Mg 1s, (**c2**) Ca 2p, and (**c3**) O 1s.

Figure 6 presents the high-resolution XPS spectra of Mg 1s, Ca 2p, and O 1s for the Mg-3Nd-2Gd-Zn-Zr alloy in the abiotic and biotic solutions. The Mg 1s spectra under all the conditions could be deconvoluted into three peaks corresponding to Mg, MgO, and Mg(OH)_2_. For Ca 2p spectra, the sample in the abiotic solution exhibited one peak attributable to CaO, while the samples in biotic solutions 1 and 2 showed two distinct peaks assigned to CaCO_3_. The O 1s spectra in the abiotic solution displayed three components representing MgO, Mg(OH)_2_, and CaO, whereas in biotic solutions 1 and 2, there was an additional peak corresponding to CaCO_3_. The XPS results were consistent with the XRD data, confirming the presence of CaCO_3_ on the alloy surface, which should have an inhibitive effect on the corrosion.

### 3.3. Corrosion Damage

**Figure 7:**
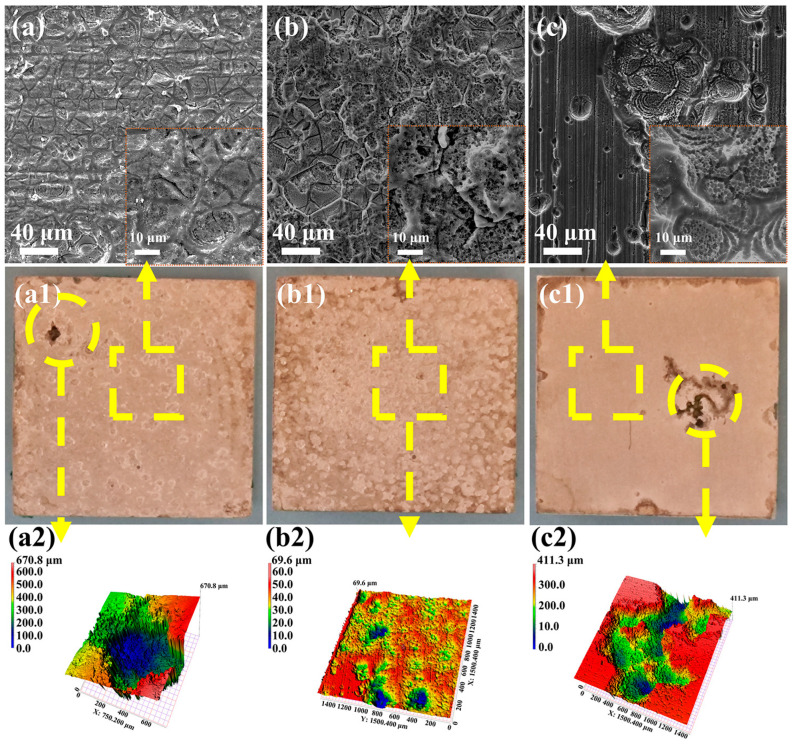
SEM images, macroscopic morphologies, and 3D color topographies of Mg-3Nd-2Gd-Zn-Zr alloy after immersion in (**a**,**a1**,**a2**) abiotic solution; (**b**,**b1**,**b2**) biotic solution 1; and (**c**,**c1**,**c2**) biotic solution 2 after removal of surface corrosion products.

Figure 7 displays SEM images, macroscopic morphologies, and three-dimensional topographies of the Mg-3Nd-2Gd-Zn-Zr alloy after immersion and removal of the corrosion products, revealing distinct corrosion characteristics among the three test solutions. The alloys in the abiotic solution and biotic solution 1 exhibited severe overall corrosion across their surfaces, while the alloy in biotic solution 2 maintained visible pre-experimental polishing marks, indicating comparatively less general corrosion. Notably, the localized corrosion damage sites on the alloy samples in the abiotic solution and biotic solution 1 were uniformly distributed, whereas the corrosion damage on the sample in biotic solution 2 was much more severe and largely concentrated in certain regions, leaving the other areas nearly unattached. In other words, a more general corrosion could be observed in most areas of the alloys in the abiotic solution and biotic solution 1, while the alloy in biotic solution 2 showed the most severe localized damage in certain areas. These results demonstrated that the low concentration of *C. vulgaris* cells could effectively inhibit the corrosion penetration while having minimal impact on the general corrosion, whereas the high concentration of *C. vulgaris* cells might significantly suppress the general corrosion but significantly promote the corrosion penetration in some local areas. The concentration-dependent dual effect of *C. vulgaris* cells on the corrosion behavior of the alloy, i.e., the low-concentration cells suppressing the localized corrosion penetration whereas the high-density cells facilitating the localized corrosion, should result from the different effects of the low and high concentration *C. vulgaris* cells on the film rupture mentioned earlier.

**Figure 8:**
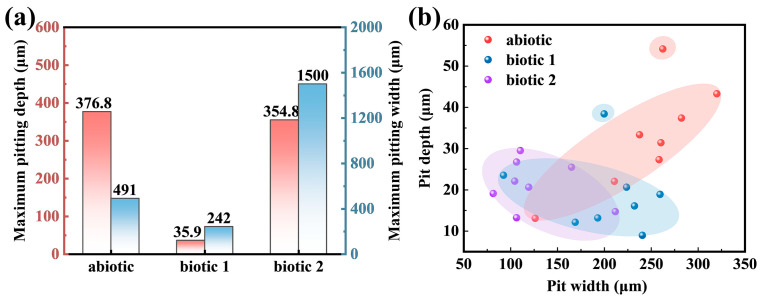
(**a**) Statistics of the maximum pitting depth and width throughout the alloy surfaces (including the concentrated corrosion damage areas) and (**b**) pitting depth vs. width in the relatively insignificantly corroded surface areas (excluding the concentrated corrosion damage areas) on the Mg-3Nd-2Gd-Zn-Zr alloy after 14 days of immersion in abiotic and biotic solutions.

The maximum pitting depth and width measurement results for the Mg-3Nd-2Gd-Zn-Zr alloy in the abiotic and biotic solutions are statistically presented in Figure 8a. In the abiotic solution, the maximum pitting depth was 376.8 μm, while in biotic solution 1, the maximum depth was only 35.9 μm. In contrast, the alloy in biotic solution 2 exhibited the most severe localized corrosion. Its maximum pitting depth was comparable to that in the abiotic solution, but its pitting damage was significantly wider (the maximum width was 1500 μm). Figure 8b presents statistical data for the pit dimensions measured at non-perforated surface locations, revealing that both the depth and width of the pits formed in the biotic solution were substantially smaller than those in the abiotic solution. Notably, the alloy in biotic solution 2 exhibited even smaller pit dimensions at non-perforated surface locations compared to that in biotic solution 1. These data demonstrated that while high-concentration algal cells exacerbated localized corrosion penetration, they simultaneously reduced the spreading of the localized corrosion.

### 3.4. Adhesion of C. vulgaris

**Figure 9:**
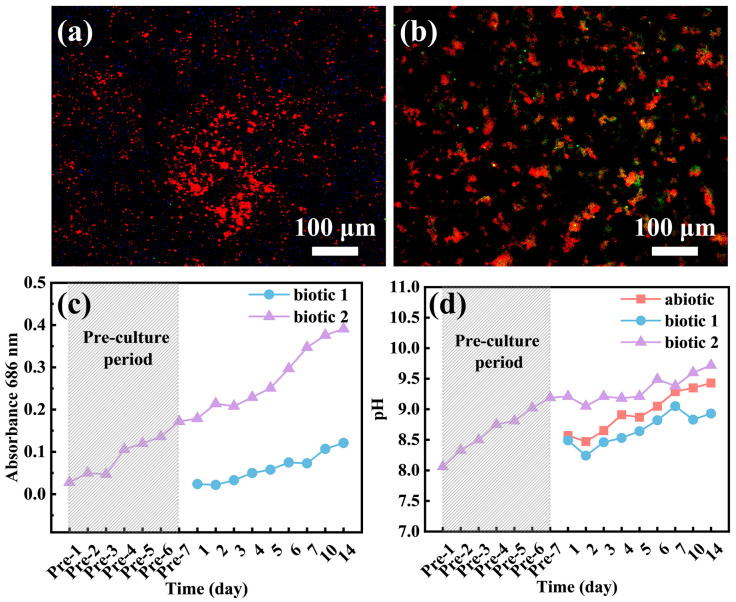
Fluorescence images of *C. vulgaris* cells on the surface of the Mg-3Nd-2Gd-Zn-Zr alloy immersed in (**a**) biotic solution 1 and (**b**) biotic solution 2 and variations of (**c**) OD values and (**d**) pH values of the solutions with time.

To determine whether *C. vulgaris* can survive in the F/2 solution containing a Mg alloy, the numbers of *C. vulgaris* cells on the surface of the Mg-3Nd-2Gd-Zn-Zr alloy and in the biotic solutions were recorded, as shown in Figure 9. Figure 9c shows the temporal variation of OD values in biotic solution 1 and biotic solution 2 containing *C. vulgaris*, which partially reflects the growth of *C. vulgaris* cells. From the figure, the population density of *C. vulgaris* cells in F/2 solution containing Mg-3Nd-2Gd-Zn-Zr alloy exhibited a gradual increasing trend over time. The figure also indicates that during the first 3 days, *C. vulgaris* was in the lag phase; from day 3 to day 10, it entered the exponential phase; and from day 10 to day 14, it reached the stationary phase but had not yet entered the decline phase. After 14 days of immersion, the morphology of the biofilm on the Mg-3Nd-2Gd-Zn-Zr alloy surface was observed using fluorescence microscopy. As under UV light, the chloroplast autofluorescence in *C. vulgaris* cells appeared red; the red dots in the images represented attached algal cells on the Mg alloy surface. The fluorescence images in Figure 9a,b showed that *C. vulgaris* cells were unevenly distributed on the surface of the Mg-3Nd-2Gd-Zn-Zr alloy, suggesting that *C. vulgaris* maintained metabolic activity on the alloy surface in the biotic solutions, which was consistent with a previous study [42]. These findings collectively indicate that the antibacterial mechanism of medical Mg alloys did not work well in the biotic solutions. Figure 9d documents temporal pH evolution, showing alkaline shifts in all the solutions. During the pre-culture stage, the metabolism of algae led to an upward trend in the pH of biotic solution 2. Biotic solution 1 exhibited lower pH values than the abiotic solution. This indirectly confirmed that the corrosion of the alloy in biotic solution 1 was inhibited, as corrosion of Mg alloys could be closely related to an increase in pH [44]. The pH of biotic solution 2 was higher than that in biotic solution 1, suggesting that corrosion in biotic solution 2 was more severe. This aligned with the EIS results, where film rupture on the alloy was observed on day 3. It is worth noting that the pH value of biotic solution 2 was significantly higher than that of the abiotic solution. This was probably because the growth of algae could promote the alkalization of the solution, which was proved by the increase in pH of biotic solution 2 during the pre-cultured stage. Although the organic acids produced by the algae would only concentrate in some positions underneath the biofilm to promote the rupture of the surface film on the alloy, they did not cause the acidification of the whole biotic solutions. The overall alkalization of the solution might result from the photosynthesis of the algae on the top of the biofilm. Moreover, the severe hydrogen evolution reaction at the rupture of surface film on the third day could also alkalize the solution. In combination with Figure 9c, the OD value of biotic solution 2 gradually increased over time and was much higher than that of biotic solution 1, indicating that *C. vulgaris* was in the stage of rapid growth. Therefore, a large number of *C. vulgaris* cells could accumulate at some local positions on the alloy surface, generating substantial organic acids through photosynthesis, which resulted in film rupture and severe localized corrosion.

### 3.5. Corrosion Mechanism of C. vulgaris on Mg-3Nd-2Gd-Zn-Zr Alloy

**Figure 10:**
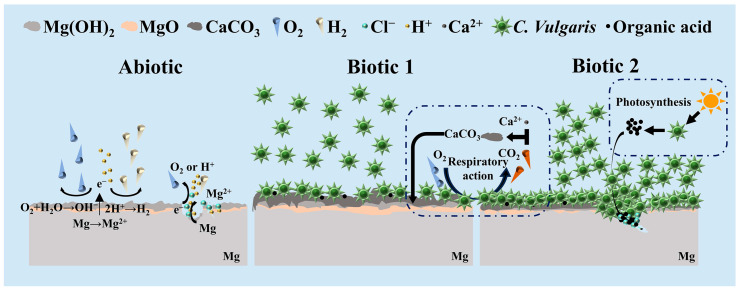
Schematic diagram of *C. vulgaris*-induced biocorrosion of Mg-3Nd-2Gd-Zn-Zr alloy.

Based on the above experimental results, the biocorrosion mechanisms of the Mg-3Nd-2Gd-Zn-Zr alloy induced by *C. vulgaris* were proposed, as shown in Figure 10. As can be seen from the figure, in the abiotic solution, the alloy primarily undergoes hydrogen evolution and oxygen reduction reactions, leading to localized corrosion. The formed Mg oxides and hydroxides on the surface provide certain protection for the substrate during the initial immersion period. The Mg oxide/hydroxide film rupture occurs later on day 7, and hydrogen evolution reaction is subsequently accelerated at the rupture sites, promoting severe localized corrosion penetration. Meanwhile, Cl^−^ is accumulated at the rupture sites, as detected by EDS, to further accelerate the corrosion.

In biotic solution 1, *C. vulgaris* cells are adhered to the surface of the Mg-3Nd-2Gd-Zn-Zr alloy to form a biofilm. As shown in Figure 8b, this biofilm exhibits a certain inhibitive effect on the corrosion of the alloy, reducing the depth and width of the localized corrosion damage. Meanwhile, due to the coverage of the biofilm, the severe corrosion penetration cannot be observed on the alloy surface in biotic solution 1 so easily as that in the abiotic solution. Moreover, the biomineralization caused by the respiratory action of *C. vulgaris* leads to a large amount of CaCO_3_ deposition on the alloy surface, as revealed by XRD and XPS, which can also effectively hinder the penetration of corrosion.

When the Mg-3Nd-2Gd-Zn-Zr alloy is immersed in the *C. vulgaris* solution that has been pre-cultured for 7 days, the surface film ruptures earlier on day 3, leading to more severe corrosion propagation than that in the abiotic solution. In a previous study, it was found that organic acids produced by *C. vulgaris* through photosynthesis were the main cause of the surface film rupture on WE43 and AM60 Mg alloys [42]. Thus, it is speculated that in biotic solution 2, the high concentration of *C. vulgaris* cells adhering to the alloy surface can also produce a large amount of organic acids through photosynthesis, which can accelerate the film rupture. Additionally, the Cl^−^ detected at the bottom of pit confirms that Cl^−^ and the high concentration of *C. vulgaris* cells synergistically accelerate the film rupture and the subsequent corrosion propagation. However, except at the rupture sites, the corrosion in other areas can be insignificant compared with that in the abiotic solution and biotic solution 1, possibly due to the accumulation of more CaCO_3_ on the surface in the latter 2 solutions.

According to the above-mentioned interpretation, it can be concluded that the lower organic acid concentration produced by the low concentration of *C. vulgaris* cells through photosynthesis can promote the formation of protective biomineralized film that effectively inhibits localized corrosion of the alloy. Conversely, the high concentration of *C. vulgaris* cells can generate concentrated organic acids at some specific sites, significantly accelerating film rupture and subsequent Mg dissolution there, while the other surface areas are still protected by the biomineralized film.

## 4. Conclusions

In this paper, the *C. vulgaris*-induced biocorrosion of the Mg-3Nd-2Gd-Zn-Zr alloy in simulated seawater was systematically investigated for the first time. Based on the findings, the following conclusions could be drawn:

(1) Although Mg alloys exhibit unique antibacterial properties in the medical field, *C. vulgaris* can adhere to the surface of the Mg alloy to form a biofilm, significantly influencing its corrosion process.
(2) Severe corrosion penetration can occur on the Mg alloy in the abiotic solution. Differently, in the biotic solution containing the low concentration of *C. vulgaris* cells, biomineralization occurs, leading to CaCO_3_ deposition on the Mg alloy surface, which to a certain degree inhibits the localized corrosion.
(3) In the pre-cultured biotic solution, the high concentration of *C. vulgaris* cells on the alloy surface may generate substantial organic acids through photosynthesis, significantly accelerating the local rupture of surface film at some sites. However, in the other areas, the surface film is intact, exhibiting good corrosion resistance compared to the films formed in the abiotic and biotic solutions.

## Figures and Tables

**Table 1:** Chemical composition of Mg-3Nd-2Gd-Zn-Zr alloy.

Element	Nd	Gd	Zr	Y	Zn	Si	P
wt.%	3.782	2.131	0.501	0.197	0.154	0.026	0.019
Element	Sn	Al	Fe	Mn	S	Mg	Others
wt.%	0.009	0.009	0.005	0.005	0.005	balance	<0.001

**Table 2:** Electrochemical parameters derived from EIS spectra for Mg-3Nd-2Gd-Zn-Zr alloy.

Media	Time (Day)	*R*_s_ (Ω·cm^2^)	*Q*_f_ × 10^−5^ (S·cm^−2^·s^n^)	*n*1	*R*_f_ (Ω·cm^2^)	*Q*_dl_ × 10^−5^ (S·cm^−2^·s^n^)	*n*2	*R*_ct_ (Ω·cm^2^)
abiotic	1	11.05	1.81	0.90	1881	94.61	0.82	1240
	3	19.29	2.52	0.86	3742	58.15	1.00	1852
	5	15.20	0.46	0.77	20.87	1.73	0.87	2552
	7	21.77	0.34	0.81	28.84	1.26	0.91	2205
	10	27.72	0.28	0.81	41.42	1.08	0.93	2952
	14	28.29	0.33	0.76	56.56	1.14	0.90	2575
biotic 1	1	10.53	1.71	0.91	2595	93.84	0.87	1288
	3	15.18	2.48	0.87	2354	122.40	1.00	852.8
	5	20.43	2.86	0.84	4159	108.40	1.00	1646
	7	24.84	3.00	0.81	6620	107.40	1.00	2037
	10	20.72	2.57	0.84	5034	136.40	1.00	1841
	14	18.78	0.30	0.90	15.46	1.17	0.92	2799
biotic 2	1	10.13	1.89	0.87	4602	42.50	1.00	2197
	3	8.87	1.16	0.79	16.29	0.79	0.93	4247
	5	12.25	0.44	0.89	21.62	1.04	0.92	3355
	7	10.86	0.42	0.86	20.75	1.17	0.93	3154
	10	11.48	0.44	0.85	19.70	1.38	0.92	1919
	14	12.22	0.50	0.83	21.28	1.49	0.93	616

**Table 3:** Electrochemical parameters of Mg-3Nd-2Gd-Zn-Zr alloy calculated from potentiodynamic polarization curves after 14 days of immersion in different media.

Media	*E*_corr_ (V vs. SCE)	*i*_corr_ (μA/cm^2^)
abiotic	−1.45	2.29
biotic 1	−1.58	7.14
biotic 2	−1.52	26.36

## Data Availability

The original contributions presented in this study are included in the article. Further inquiries can be directed to the corresponding authors.
